# Secondary Angle Closure Glaucoma in Weill–Marchesani Syndrome

**DOI:** 10.3390/diagnostics14202303

**Published:** 2024-10-16

**Authors:** Valeria Coviltir, Miruna Gabriela Burcel, Maria Cristina Marinescu, Bianca Maria Urse, Ciprian Danielescu

**Affiliations:** 1 Ophthalmology Discipline, Carol Davila University of Medicine and Pharmacy, 050474 Bucharest, Romania; 2 Clinical Hospital for Ophthalmological Emergencies, 010464 Bucharest, Romania; 3 Brasov County Emergency Clinical Hospital, 500326 Brașov, Romania; 4 Faculty of Medicine, Transilvania University of Braşov, 500036 Braşov, Romania; 5 Physiology III Discipline, Carol Davila University of Medicine and Pharmacy, 050474 Bucharest, Romania; 6 Ophthalmology Discipline, Surgery II Department, Grigore T. Popa University of Medicine and Pharmacy, 700115 Iași, Romania

**Keywords:** Weill–Marchesani syndrome, microspherophakia, glaucoma

## Abstract

We report a case of a 16-year-old girl presenting to our clinic with decreased visual acuity and increased intraocular pressure in both eyes. The ophthalmological examination revealed best-corrected visual acuity (BCVA) of 0.3 in the right eye (R.E.) and 0.4 in the left eye (L.E.) and intraocular pressure (IOP) of 46 mmHg in the R.E. and 42 mmHg in the L.E., with a 360° closed angle on gonioscopy, pupillary block due to bulging, a hyper-spherical lens and high corneal thickness, without ectopia lentis or cataract. The eyes responded poorly to pharmacological mydriasis; therefore, the lens equator could not be visualised. The patient had a history of pulmonary stenosis, short stature and no significant cognitive deficits. These elements point to the diagnosis of Weill–Marchesani syndrome, and the ophthalmological management was surgical, including lens extraction and the installation of a capsular tension ring, an intraocular lens and a Shunt ExPress implantation. Evolution was favourable, with improved BCVA of 0.7 in the R.E. and 0.63 in the L.E. and IOP of 14 mmHg in the R.E. and 13 mmHg in the L.E., without topical or systemic treatment at the 6-month follow-up. Weill–Marchesani syndrome has a complex presentation, with ophthalmological, musculoskeletal, cardiac and psychiatric manifestations. Usually, this leads to a need for a multidisciplinary approach. The ophthalmologic symptoms are often the cause of presentation to a specialist, and glaucoma is the most threatening of the ocular pathologies, with possible evolution into irreversible blindness; therefore, prompt surgery and careful follow-up become key components of the treatment plan. As a take-home message, we encourage a high degree of suspicion of Weill–Marchesani syndrome in such cases.

Weill–Marchesani syndrome is a rare genetic disorder of connective tissue characterised by short stature, restricted articular movements, brachydactyly, cardiac abnormalities and eye anomalies including microspherophakia, ectopia lentis, increased corneal thickness, severe myopia and secondary glaucoma [1,2]. There are different mutations depending on the mode of inheritance. Autosomal dominant (AD) inheritance is caused by a heterozygous mutation within the fibrillin-1 gene (FBN1) [3,4]. Autosomal recessive (AR) cases can be caused by diverse mutations that include the genes for the ADAM metallopeptidase with thrombospondin type 1 motif 10 (ADAMTS10) and motif 17 (ADAMTS17), and for the latent transforming growth factor beta binding protein 2 (LTBP2). The prevalence is estimated at 1 in 100,000 in the population [1,5]. Most cases have been described by ophthalmologists due to the characteristic ocular signs and symptoms, even though on initial exams many can be misdiagnosed as high myopia and angle closure glaucoma [1,3] (Figure 1 and Figure 2).

Also known as microspherophakia–brachydactyly syndrome, Weill–Marchesani syndrome is a rare genetic disorder of the connective tissue. In a study published by Faivre et al., the autosomal dominant (AD) and recessive (AR) mode of inheritance accounted for 39% and 45% of the cases, whilst the remaining percentage were sporadic cases [4,5]. Our clinical findings are similar to those in the literature, with the exception of ectopia lentis, cataract, joint restriction (which was not evaluated by a specialist) and mental retardation. The characteristic features of Weill–Marchesani syndrome are as follows: microspherophakia (84%), myopia (94%), ectopia lentis (73%), glaucoma (80%), cataract (23%), short stature (98%), brachydactyly (98%), joint restriction (62%), cardiac abnormalities (24%) and mental retardation (13%) [3,4]. Recently, another ophthalmologic characteristic has been described, which is the abnormally increased corneal thickness that was also found in our case [6,7]. The increase in corneal thickness is associated with the activation of keratocytes in the anterior stroma, suggesting that corneal thickness increase can be a newly described feature of Weill–Marchesani syndrome [8].

The morphological characteristics of Weill–Marchesani syndrome lead to multiple clinical manifestations and necessitate a specific surgical approach. The abnormal spherical shape of the lens (with an increased anterior curvature - and, therefore, increased refractive power) and the anterior displacement of the lens (causing a refractive shift) contribute to decreased visual acuity [9,10]. This can often be misdiagnosed as simple high myopia. The increased lens thickness along with the weak zonules allow the lens to move forward and cause pupillary block that leads to secondary closed-angle glaucoma [9].

Besides Weill–Marchesani syndrome, mutations in the LTBP2 gene can also cause congenital glaucoma [1], a rare disease with a high risk of blindness in children and which may also be accompanied by systemic pathologies [11].

The extremely high IOP associated with this pathology leads to severe optic nerve damage if the diagnosis and treatment are not fast and efficient. Thus, lens extraction and intraocular lens implantation is the correct approach in secondary closed-angle glaucoma with pupillary block in microspherophakia. In our case, the absence of angle elements visible on indentation gonioscopy determined the decision to implant a filtration device (Shunt ExPress) in order to ensure effective IOP control. In the literature, laser iridotomy has been described as a treatment option, but disease progression is not necessarily managed by it, ultimately leading to lens extraction [9]. Trabeculectomy without lens extraction was also described in the literature, and it was associated with an increased risk of malignant glaucoma, especially in the presence of a shallow anterior chamber [9,12], like in our patient.

Weill–Marchesani syndrome presents with a variety of clinical features of an ophthalmological, musculoskeletal, cardiac and psychiatric nature. This peculiarity implies the need for a multidisciplinary approach. The ophthalmologic symptoms are often the first reason for a patient consulting a specialist, and glaucoma is the most threatening of the ocular pathologies, with possible evolution into irreversible blindness; therefore, surgery becomes the key treatment. Our take-home message is that clinicians should have a high level of suspicion of Weill–Marchesani syndrome in cases such as this with high lenticular myopia, multiple lens anomalies and angle-closure glaucoma in young patients, all associated with several systemic manifestations.

## Figures and Tables

**Figure 1:**
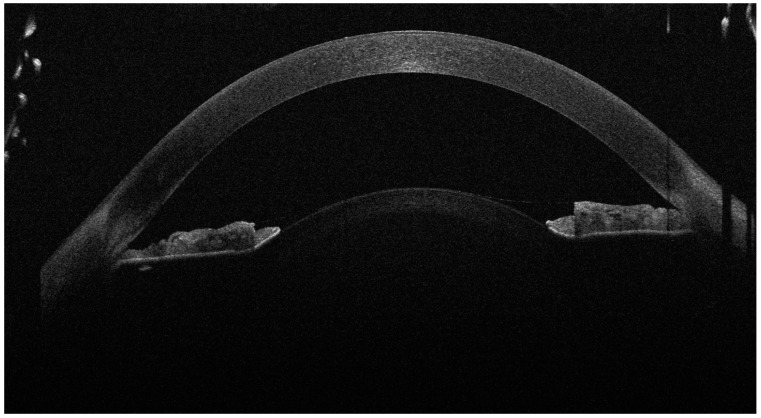
The anterior OCT aspect of the right eye, showcasing high corneal thickness, iridocorneal contact and a high lens vault (the anterior face of the lens bulging forward through the pupil). As the left eye aspect was highly similar, we chose to showcase just the right eye for brevity. A 16-year-old female presented to our clinic with decreased visual acuity (VA) and increased intraocular pressure (IOP). The patient was diagnosed with juvenile glaucoma 2 months ago and high myopia since childhood, and was currently under topical antiglaucoma treatment with dorzolamide, brimonidine and timolol. Her best-corrected visual acuity (BCVA) on the Snellen chart was 0.3 (20/66) with −14.0 D in her right eye (R.E.) and 0.4 (20/50) with −14.0 D in her left eye (L.E.). The intraocular pressure (IOP) at presentation was 46 mmHg in the R.E. and 42 mmHg in the L.E. On examination, the anterior chamber of both eyes was very shallow, and the iris was bulging forward. On gonioscopy, the angle was closed, with 0 elements visible even on indentation. Posterior segment investigation revealed a cup-to-disc ratio (C/D) of 0.4 in the R.E. and 0.3 in the L.E., without any myopic changes on the retina. The ocular biometry showed an increased lens thickness of 4.80 mm for the R.E. and 4.71 mm for the L.E. and an axial length of 22.26 mm (R.E.) and 22.52 mm (L.E.), values that could not be correlated with the high myopia. The anterior segment OCT also revealed an increased anterior curvature of the lens along with an increased corneal thickness of 683 μm in the R.E. and 674 μm in the L.E. After instilling tropicamide 1% and phenylephrine 10%, the pupil was still poorly dilated, with no visualisation of the lens equator. The general physical examination noted a short stature (1.44 m) and brachydactyly. The patient had undergone cardiac valve surgery at the age of 10 after being diagnosed with pulmonary valve stenosis. The patient had normal cognitive function. No significant heredocolateral data were described by the patient or the parents. Corroborating the ocular examination and systemic manifestations of the patient, the diagnosis of Weill–Marchesani syndrome was made. The patient had not undergone genetic testing and declined all tests due to financial reasons. In order to prevent the ongoing damage of the high IOP, the patient underwent surgery on both eyes 1 week apart. The surgical procedure involved lens extraction though phacoemulsification, using the stop-and-chop technique, followed by installing a tension ring in the capsular bag; an intraocular lens implantation (Alcon, type AcrySof MA60AC, refractive value of +22.0 D for the right eye and +22.5 D for the left eye) and a Shunt ExPress implantation—a non-valved device that connects the anterior chamber with the intrascleral space through a partial thickness scleral flap.

**Figure 2:**
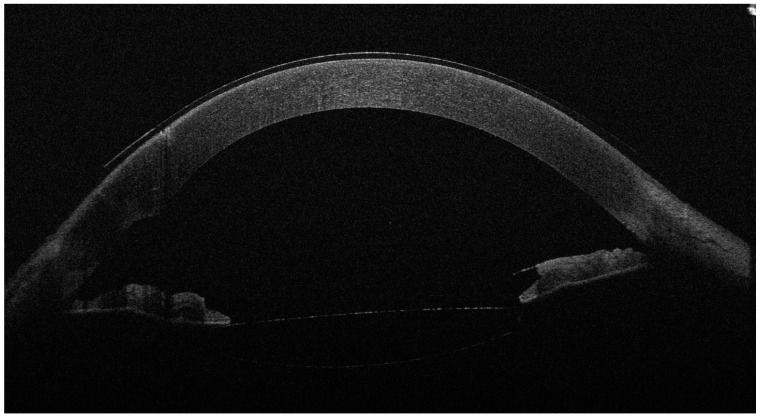
The anterior OCT aspect of the right eye 6 months after surgery, with an open iridocorneal angle, a deeper anterior chamber and a well-positioned intraocular lens in the posterior chamber. As the left eye aspect was highly similar, we chose to showcase just the right eye for brevity. Slit-lamp examination also revealed the well-positioned shunt and normal IOP without treatment (14 mmHg in the R.E. and 13 mmHg in the L.E.). The BCVA was 0.7 (20/28) with −0.50 D in the R.E. and 0.63 (20/30) with −0.50 D in the L.E. In the second figure, the corneal epithelium is visibly thicker than before surgery. While corneal edema is a known complication of cataract surgery, the slit-lamp aspect of a clear cornea, the good visual acuities and the long duration of follow-up after surgery (6 months) argue against epithelial thickening as a phaco complication. However, confocal microscopy examination of a Weill–Marchesani case in the literature revealed abnormally shaped keratocytes with a higher density in the anterior stroma and normal morphology and density in the posterior stroma [6]. This may suggest that the anterior stroma and superjacent corneal epithelium are more vulnerable to stressors and may respond by water accumulation and edema; however, more studies are needed to explore this characteristic of the syndrome.

## Data Availability

All relevant data have been presented in this manuscript, and further inquiries can be directed to the corresponding author.
